# Family Caregivers’ Trajectories of Distress While Caring for a Person With Serious Mental Illness

**DOI:** 10.1177/10497323231203627

**Published:** 2023-10-31

**Authors:** Anne Martha Kalhovde, Gabriele Kitzmüller

**Affiliations:** 1 423628 Jæren District Psychiatric Center, Bryne, Norway; 2 UiT, The Arctic University of Norway, Narvik, Norway

**Keywords:** distress, family caregiving, hermeneutics, serious mental illness, safety, unpredictability

## Abstract

Serious mental illness (SMI) can significantly impact the lives of individuals and their families. These families often experience great emotional distress over time due to the early onset of SMI, which in turn leads to long-term trajectories and only partial recovery. However, we do not fully understand the emotional distress of family caregivers. Thus, our aim was to enrich the understanding of the lived experiences of family caregivers’ emotional trajectories of distress while caring for persons with SMI. We conducted a secondary analysis using a hermeneutic approach to the narratives of seven family caregivers from a study on living with voices unheard by others. Participants’ trajectories of emotional distress came forth as being thrust on an unpredictable, intensely worrisome, and indefinite journey. The following themes highlighted this tumultuous journey: *fumbling in the dark trying to grasp the incomprehensible, “on your toes”—enduring unpredictability, facing different forms of fear,* and *battling waves of sadness and regret.* Caregivers face multiple threats to their well-being and sometimes even to their health. Their distress appeared to vary according to their relationship with the person with SMI, whether they lived with the ill person, illness trajectory, and amount of violent or suicidal behavior. The results underscore the need for individualized and timely information, opportunities for dialogue with healthcare providers with and without the person with SMI, and inclusion in care planning. Caregivers who have experienced trauma, threats of violence, and rejection require special attention.

## Background

In this study, we aimed to enrich the understanding of the lived experiences of family caregivers’ emotional trajectories of distress in caring for persons with serious mental illness (SMI). SMI can profoundly affect families. Today, family members increasingly provide daily practical, emotional, social, and financial support to relatives with SMI (Kamil & Velligan, 2019). Family caregivers of individuals with SMI appear to have a higher caregiving burden (Hastrup et al., 2011). Long-term or repeated shorter periods of informal caregiving are associated with more symptoms of psychological distress and risk of poorer mental health (Lacey et al., 2019).

Flyckt et al. (2013) found that family caregivers perceived the mental burden of caregiving to be the most problematic aspect. Caregivers of individuals with SMI have more depression, anxiety, and physical health issues than those of individuals with other serious long-term illnesses and healthy controls (Kamil & Velligan, 2019). More protracted and severe SMI often leads to greater perceived caregiving burden (Siddiqui & Khalid, 2019). Caregivers’ emotional distress often involves embarrassment and self-directed emotions such as shame, guilt, self-blame (Awad & Voruganti, 2008; Wasserman et al., 2013), grief (Rachamim et al., 2022), hopelessness, helplessness, and fear for their loved one and their own situation (Ntsayagae et al., 2019). Family disruption, conflict in interpersonal relationships, and stigma (Shiraishi & Reilly, 2019) are closely related to caregiving in families with SMI. Although caring for persons with SMI can be rewarding, the burden of care can overshadow the positive aspects and significantly reduce the caregivers’ quality of life (Shiraishi & Reilly, 2019).

Knowledge of the patterns and meanings of family caregivers’ emotions may enhance empathic responses and increase emotional support from healthcare professionals (Shiraishi & Reilly, 2022). A better understanding of family caregivers’ distress trajectories may enhance healthcare providers’ ability to identify caregivers’ specific requests and adapt healthcare and nursing interventions to their individual needs.

## Methodology

The methods are grounded in the philosophical hermeneutics of Gadamer et al. (2004) and inspired from suggested steps by Fleming et al. (2003) for Gadamerian-based nursing research: (a) deciding on a research question, (b) gaining understanding through dialogue with participants, (c) gaining understanding through dialogue with texts, and (d) establishing trustworthiness. According to Gadamer et al. (2004), true dialogues typically contain open questions in which those involved attempt to embrace the possibilities that lie in the answers, not merely confirm what they already know. To gain a new understanding or reach beyond the limits of our own suppositions, we must expose ourselves to opposing views (Gonzales, 2006). When research partners engage in true dialogue and opposing views, their preunderstandings can be adjusted or countered, leading to new sources of knowledge, and their horizons of understanding may merge (Gonzales, 2006).

We performed a secondary analysis of preexisting primary data derived from a hermeneutic and phenomenological study (Kalhovde, 2015). According to Hinds et al. (1997, p. 409), a qualitative secondary analysis holds the potential to provide important knowledge from descriptively rich yet underused qualitative data sets. This approach enables researchers to pose new research questions to existing data, thereby achieving a new understanding or analytic expansion as termed by Thorne (1998). Secondary analysis allows for the involvement of new researchers (Heaton, 2008). Thus, data that were underemphasized in the primary study may be investigated from new perspectives (Ruggiano & Perry, 2019). Secondary analyses also decrease the probability of existing data sets being underused (Thorne, 1998) and may prevent research fatigue among participants (Ashley, 2021).

## Methods

In the present study, the first author, who collected the original data, invited the second author to re-analyze the anonymized transcribed interviews of the family members of individuals with psychotic disorders and experiences of auditory hallucinations. The second author posed questions based on her experience as a nurse providing psychosocial care to patients and their families and research on family members of people with chronic illness. This was aimed at understanding and challenging the first author’s preunderstandings based on her in-depth knowledge of the context, having conducted the interviews, in addition to her experience of clinical mental health nursing practice.

The primary data derived from a study of peoples’ experiences of living with voices that others could not hear (Kalhovde et al., 2013, 2014). The interviews were analyzed with the explicit aim of enhancing the understanding of the lived experience of hearing voices. Family members confirmed that ill relatives had shared little of their experiences of hearing voices with them, and a separate publication of their experiences was therefore omitted from the primary project. To do justice to these individuals, we decided to explore their narratives regarding their emotionally laden experiences as caregivers and formulated the following research question:

How do family caregivers experience emotional distress when caring for people with SMI over time?

### Participants

In the primary study, the first author recruited participants through various channels. Some voice hearers from the main study gave the first author permission to contact one or two immediate relatives. A local newspaper article informed the public about the study and the need for additional participants. A local affiliate of the national advocacy group of Mental Health Carers Norway (LPP) assisted the first author by sending written information about the study to all members and hosting a meeting where the first author presented the research project. Seven relatives from seven families responded: three mothers, one father, one spouse, and two siblings (four women and three men). They all met the criterion of being adult relatives of a person with a psychotic disorder who had repeated experiences of hearing voices or sounds they had heard alone for at least one year.

The participants’ ages ranged from late 30s to early 70s (median age, 40 years). Six were the main caregivers, and three were married. Three lived with the ill relatives. Six were employed, and one was a pensioner. All but two participants cared for children. Relatives with SMI (*n* = 8) aged 19–30 years (median age, 30 years), four women and four men, had been diagnosed with SMI for approximately 4 to over 10 years (six had a schizophrenia spectrum diagnosis, one had psychotic depression, and several had co-occurring substance use and posttraumatic stress disorder). All participants reported that their relatives with SMI had used or were still using neuroleptic medications and had several involuntary hospital admissions. Most received a disability pension, four lived in supported housing, and two were part-time students.

### Interviews

In the primary study, the first author conducted interviews by asking the participants to convey their experiences related to a family member who heard voices. Most answered this question by elaborating on the challenges of caring for seriously ill relatives. The first author posed clarifying questions and summarized statements to ensure mutual understanding. Immediately after the interviews, the first author noted the contextual information, first impressions, reflections, and questions. Five of the interviews were conducted at the first author’s workplace and two at the interviewees’ homes. The interviews lasted from 80 to 120 minutes. Before the first author validated the transcripts, a research assistant transcribed the digitally recorded interviews verbatim.

### Analysis

We analyzed the transcripts of the family members (*n* = 7) based on Gadamer et al. (2004) descriptions of hermeneutic circling and the concretizations suggested by Fleming et al. (2003). After reading the transcripts several times, we wrote down expressions that illuminated the underlying meaning of the entire text in relation to the research question. We then compared, challenged, and revised the manuscript accordingly. Next, the second author examined and noted the sentences and sections and formed written interpretations of the emotional trajectory of the participants. These were challenged by the first author, and the second author formed the preliminary themes and subthemes, which the first author challenged again. Third, the themes and subthemes were compared with the overall written understanding and revised through an iterative spiraling process. We concluded the analysis when our understanding of the parts coincided with our understanding of the entire text, and we achieved an expanded and shared understanding. Finally, illustrative quotes were chosen.

### Ethical Considerations

Ethical approval for the original study was granted by the Regional Committee for Medical and Health Research Ethics and the NSD data protection services (P REK NORD 118/2006, NSD 15313). All research procedures in the original study and secondary analysis complied with the Helsinki Declaration. All the participants provided written informed consent to participate in the primary study. Only anonymized data were used in the secondary analyses, and additional steps were taken to avoid participant identification.

### Trustworthiness

We detailed the research process and findings to promote transparency and trustworthiness (cf. Fleming et al., 2003). The interviews were conducted by the first author in a dialogical manner with the aim of achieving mutual understanding (cf. Fleming et al., 2003), albeit with only one interview with each participant. Conducting several interviews or receiving feedback from the participants would not necessarily have increased the quality of the data because of the participants’ ever-transforming preunderstandings (Fleming & Robb, 2019). The interviews were sufficiently detailed and varied to enhance understanding of the phenomenon in question. The quality of the transcripts was confirmed by the first author. The first author’s involvement in the interviews and notes taken immediately after them made it possible to consider contextual information and participants’ emotions.

In our efforts to refute inaccurate understandings and acquire new knowledge, we confronted our preunderstandings through dialogues (cf. Gadamer et al., 2004) with each other, fellow researchers, colleagues, and other family caregivers. Through these dialogues and presentations of preliminary findings, we also sought to validate and confirm the actuality of the results. The analysis was carried out through prolonged engagement with the texts and close collaboration between researchers to present the sample in a way that made it possible for the readers to decide whether the findings were applicable to their own context without compromising the anonymity of the participants. We sought credibility by ensuring that the participants’ perspectives were represented clearly and truthfully (Lincoln & Guba, 1985).

## Results

Analysis of the narratives revealed that family caregivers’ trajectories of emotional distress in caring for a person with SMI could be understood as being thrust toward an unpredictable, intensely worrisome, and indefinite journey. The following themes highlighted the emotional aspects of this tumultuous journey: *fumbling in the dark trying to grasp the incomprehensible, “on your toes”—enduring unpredictability, facing different forms of fear*, illuminated by three subthemes, and *battling waves of sadness and regret.*

### Fumbling in the Dark Trying to Grasp the Incomprehensible

The participants struggled to make sense of their family members’ initially altered behaviors and shifting moods. First, they were concerned about subtle changes. Eventually, they became increasingly bewildered and shocked by self-harm, suicide attempts, incomprehensible allegations, and behavior and did not know how to react. In their attempts to understand their loved one, most described having to piece together information from things their loved one said and did, along with information from healthcare professionals. Young adults with SMI refused to explain why they withdrew, hurt themselves, attempted suicide, or avoided confiding in their family members:

She didn’t want to talk about her difficulties at home. No. To this day she still hasn’t told me that she hears voices, but she did tell me to read a book [on first-hand experiences of psychosis]. I read it and she said: “That’s how my life is,” […] then I understood how she was.

Feelings of anguish and resignation arose because of rejection by their loved ones for shorter or longer periods. One parent recounted that their son had refused to have anything to do with them for years:

Then, for some reason or other, he decided to cut us off, totally. Several years passed. […] Can you imagine how it felt when I was attending a meeting and knew that 100 meters from here there’s someone you love who doesn’t want to see you? It was awful […] a kind of feeling of resignation, very often despair.

The caregivers regretted receiving little and unhelpful advice from healthcare professionals. One parent was encouraged by a healthcare provider to read a biography to understand the child better. Instead, she was devastated by the prospect that her child might have had a similar 20-year struggle to that of the author. Eventually, the participants related the troublesome actions of their loved ones to mental illness: “I don’t think it’s [name] doing these things to me […], because [name] isn’t like that, it’s the illness.”

Similarities between ill relatives and other family members with serious mental illnesses amplified their worries about the current situation.

Grandma wanted to kill one of her children because she thought he had caused her illness, he had to be cared for, away from home. This memory made me fear that my own daughter could harm her brothers and sisters.

For one of the parents, who had experienced hallucinations herself, it was easier to grasp what the child’s experiences were about, but she still wondered: “It was so real to me [the parent’s own hallucinations]. If they experience things like that, I can understand that they get angry, scared or amused.”

In the acute phases, participants were confounded, worried, and disempowered by their relatives’ strong emotions and shocking behaviors. Being a witness to their loved one’s experiences of hearing voices, seeing things they experienced alone, or delusional and persecutory beliefs were deeply worrisome and perplexing:

I think these voices, to put it bluntly, are a pain in the ass […] telling you to cut your head off or whatever they might say, that you’re nobody and that you should go and kill yourself. […] I can see when the voices are gone and when they return. Then she sits and giggles for herself and I don’t like that. I get a bit stressed and pissed off at her.

The mothers voiced despair, impatience, and frustration during the phases in which their loved ones kept to themselves asleep most of the day and were unengaged without initiative.

### “On Your Toes”—Enduring Unpredictability

The participants’ daily lives were constantly disrupted. Numerous sudden, dramatic episodes rendered them constantly vigilant for signs of relapse: “I feel that these relapses are hard to deal with, it’s barely half a step forward and two steps back. […] How long will it last before it’ll get worse again?” For the family members who lived with the ill person, it was challenging for weeks, months, and even years at a time to live a normal day-to-day life and to make short- and long-term plans. Gradually the participants recognized patterns and were less bewildered, albeit persistently alert.

In the acute stages of a loved one’s illness, frequent and often involuntary psychiatric care was necessary. The participants had to be prepared for contact with healthcare services day and night. Some had to be ready to contact the police for assistance. Mothers reported having difficulty sleeping due to fear of self-harm or suicide attempts that often occurred at night.

The thing I find so scary about it […] is that it often happens at night. It starts in the evening and at night when we’re supposed to relax, when we’re going to sleep, and we know that sometimes she’s suffering really bad.

Family members reported being on their toes while at work and having to leave at short notice: “I haven’t told my co-workers everything, but they know the most important things. So, when I call them and ask them to step in for me, they do it if they can.”

Physical sensations related to recurrent self-harm came forth:

When I felt my heart pounding, I thought: Oh no! Is it that stuff again? Here we go again. […] What am I gonna do? […] So, you’re scared. You kinda got scared that this time, this time she’ll manage to commit suicide. […] So it’s like you never got a break from that.

Not being understood or taken seriously by the police and healthcare services was frustrating. They assisted or witnessed admission to the hospital but often doubted whether the information they provided was noted. Being left in the dark by healthcare providers withholding valuable information during hospital stays intensified family members’ distress. However, they still had to take care of their loved one after discharge despite the lack of information about the illness and medication:

Suddenly the psychologist told me, well, you have a very sick daughter. […] I asked, “What are you referring to?” Yeah, she couldn’t say more than that, because of confidentiality. […] So, we went on holiday with new pills, not knowing anything about the side effects […].

### Facing Different Forms of Fear

Fear dominated the family members’ accounts of their caring trajectories, albeit at various levels and for various reasons, as described in the three subthemes.

#### Worrying About Their Family Member

Family caregivers’ most prominent fear was that their loved one could harm or kill themselves. Suicidal attempts and episodes of self-harm had occurred multiple times in most of the families: “I guess we have to live a normal life as far as we can, but every time she runs to the bridge and every time the police show up it’s hard.”

Then he’d emptied that bottle plus the entire blister pack of pills. He could barely manage to stand properly and went into the kitchen. I grabbed him by the shoulders and asked: “What’ve you done?” I wanted him to tell me. He just opened his mouth, and I saw that his teeth were full of crushed pills.

Participants did not always rely on healthcare professionals’ assessments. Premature discharge from mental health facilities increased the emotional burden on family members if the person still had suicidal thoughts:

A lot of the times when she came from [psychiatric hospital], they just called me and said: “She’s on the boat on her way home now.” What! When she came home, she told me: “I nearly jumped off the boat […].” She wasn’t well enough to leave the hospital.

The participants were concerned about the quality of treatment and the extensive use of antipsychotic medication. Some worried that their family members might reject the treatment, or harm themselves, due to insufficient safety measures: “I’m scared, because there’s been a lot of testing of medication, and I can see that it’s really messing up her body. It makes me so worried.”

Their constant worries about the well-being of their family members and their actions to prevent self-harm consumed most of the participants’ energy and put their own and other family members’ lives on hold for extended periods.

#### Being Concerned About the Harmful Influence on Others

Two mothers expressed concerns about the influence of their daughters’ illnesses on other children in the family. One said:

She did tell [name] a whole lot, he was young, you know, just six years old […] She told him she’d broken a mirror and had cut herself […] and then she showed him […]. Last winter he stressed alot about his arms, he couldn’t stand wearing short sleeves, he had to wear long sleeves.

Another mother tried to deal with the influence of her daughter’s illness by avoiding the topic within the family:

We never talk with the other children about it, for example, we don’t use the name of the hospital she’s in, because that word has a very negative meaning. We say she’s in hospital and that she’s sick […] we talk as little as possible. The thing we’ve found about openness […] people almost get scared.

Sometimes the self-destructive behavior of the person led to potentially dangerous situations, threatening the safety of others: “Suddenly he’d pull the handbrake and jump out of the car when it was going at 60 or 70 [kph] and in heavy traffic, because he was so scared that we were planning to kill him.”

#### Terrified of Being Attacked

Two parents experienced episodes of severe violence in which they were afraid their sons would seriously injure or kill them:

He came after me and tried to strangle me. He’s a lot bigger than I am, and his weight gives him strength. I was scared, terribly frightened and called for help. It was bad and continued like that, so when I called the doctor, he said: “This can’t go on.”

Being afraid of their child was a painful and conflicting experience. Living together meant that one mother had to tiptoe through life for fear of triggering delusional beliefs and attacks.

I prepared meals very carefully, yeah, without a sound. Vacuumed when he was out or cleaned the house […] when he was on the Internet. So, then he was occupied with that, so I could do housework and stuff. […] He wasn’t out that much.

In this case, there seemed to be a vicious circle of fear in which he attempted to protect himself from his mother while turning her life into a frightening existence. Despite her desperate fear of him, she was reluctant to seek help because of his fear of being admitted to the mental healthcare department. When she eventually sought help, neither the police nor healthcare services responded quickly enough to prevent him from injuring her or damaging their home. Although he had received coordinated healthcare for several years, she continued to be wary of new attacks and financial abuse.

### Battling Waves of Sadness and Regrets

The parents frequently expressed feelings of guilt, shame, grief, and anger. They expressed deep concern about their loved one’s future prospects and were saddened by the person’s loss and lack of opportunities. The lack of hope in these participants’ accounts was prominent, although several mentioned the importance of “finding something to hope for.” Seeking answers in the past was a means of explaining why their loved ones had fallen ill. They talked about waves of regret and guilt in relation to past incidents, things they had not been aware of, or had possibly misunderstood. All described guilt for not having done enough for their loved ones and promoting their recovery.

Parents reflected on a multitude of possible reasons for their ill child’s troubles, such as a prolonged birth process, lack of understanding of the magnitude of behavioral changes during adolescence, and incompetent school nurses, doctors, or child welfare staff. Abuse occurred in two families. Others pondered whether abuse had occurred without their knowledge.

I blamed myself for having missed something when she was growing up, I searched for invisible things. When she had school problems, I thought someone had done something to her, some abuse within the family that I had overlooked. I felt a lot of guilt.

Their family member’s illness called for deep and painful emotions and helplessness. Getting angry with the ill person was perceived as shameful: “Sometimes she seems distant and usually you expect answers […]. Many times I’m annoyed but afterwards I know that it wasn’t right to react like that […] probably her voices were bothering her.”

Mothers revealed deep feelings of despair and grief about being unable to help their children or not receiving appropriate help from the healthcare system:

The first two years felt like living in hell. […] People who should have helped us became our enemies. They didn’t show understanding or empathy […]. Sometimes it was so devastating that I thought: “Oh my God, I’ll kill both of us. It’s the easiest way out of this […].”

It was hard to accept that their loved ones’ illnesses would last a long time, possibly for life. Feelings of hopelessness came forth: “They said there was no hope, it was hell [to hear] […] they probably wanted to confront us with reality […] we were deep down already, and this pushed us even further down.”

Parents could hardly bear the phases in which their family members, consumed by delusions, withdrew or rejected them, especially if they believed them to be a threat:

She saw something horrifying in my face and she thought I was sinister and ugly. She was so scared of me. […] It was a pretty dreadful experience […] suddenly I understood I couldn’t help her. […] It felt like my body was being torn apart, it felt like I was bleeding inside, and it hurt, it really hurt […]. Now I realize what it means to have your heart wrung out.

One parent wondered about blame and being the cause of their son’s illness:

You ask yourself what’s the cause and what’s the effect? Is it something to do with me, am I to blame for his development? […] Has my background had any influence? Because it must be connected to how I behaved with my family.

Some parents were unable to share their caring experiences with others due to shame or a lack of understanding, while siblings confided with others. Feelings of sadness and a bad conscience for not being able to do enough were common among family caregivers, although siblings were not as affected by these emotions. One of the siblings spoke of her personal growth due to her experience as a caregiver, despite all the hardships.

## Discussion

The findings showed that family caregivers’ emotional trajectories of distress in caring for persons suffering from SMI were thrust toward an unpredictable, intensely worrisome, and indefinite journey. The intensity of caregivers’ distress varied according to their relationship with the person with SMI, whether they lived together with the ill person, instability of the illness trajectory, and the amount of challenging behavior, such as violence or suicide attempts. Parents seemed to be more exposed to violent behavior and despair over not knowing how to or being unable to help. The quality of communication between caregivers and ill family members and between caregivers and healthcare workers also appeared to be closely related to caregivers’ distress. The trajectories entailed a long, turbulent journey much like being on a never-ending and unpredictable rollercoaster ride with the ill family member as the center of gravity in the family, as reported by Weimand et al. (2020).

The participants reported highly stressful experiences of struggling in the dark with their loved ones’ incomprehensible reactions and actions. They repeatedly did not know what to think about, feel like, or do in situations that shifted from fairly coherent and predictable to chaotic and disordered, as Karp and Tanarugsachock (2000) reported. Even when things were going well, the participants had a lingering uncertainty regarding how long it would last and constant preparedness for upcoming crises. These findings are consistent with those of Weimand et al. (2020) and Wiens and Daniluk (2009). Participants’ preconceived notions based on prior dire experiences of familial mental illness appeared to reinforce the anticipation that similar devastating experiences awaited them. Family caregivers’ belief that the illness is long term, and the consequences are severe and out of their control tends to lead to negative appraisals of care, which are closely related to distress (Jansen et al., 2015a).

Our findings show that caregivers unsuccessfully sought explanations from their loved ones and received insufficient and delayed information and guidance from professionals, as reported by Landon and Shepherd (2016). Thus, receiving information about the diagnosis was of little emotional comfort, although it allowed some participants to put their loved ones’ unsettling behavior into a broader context. Participants’ feelings of hopelessness were reinforced when the diagnosis was passed on without empathy by healthcare workers or if they predicted a negative future development of the illness. In addition, family caregivers in a study by Outram et al. (2015) experienced a lack of basic communication skills among mental health professionals when breaking difficult news. They missed empathic listening, respect, and validation of their feelings and felt excluded from the medical care process.

Furthermore, the participants described having to intervene against their family members’ will and sometimes behind their backs as emotionally draining. Most experienced long phases of rejection and felt alienated and abandoned by mental health professionals. Healthcare providers seemed to contribute to a tug-of-war between parents and their children with SMI by avoiding information sharing owing to confidentiality. In line with these findings, Doody et al. (2017) concluded that the involvement of family caregivers in care planning needs improvement and that issues of confidentiality should be addressed to facilitate the exchange of information and shared decision-making. Healthcare interventions aimed at improving communication with and within families with SMI and resolving conflicts are often necessary and beneficial (Bergström et al., 2018; Shiraishi & Reilly, 2022), but are poorly implemented (Hestmark et al., 2021). Participants’ vigilance around the clock also led to prolonged periods of disturbed sleep and exhaustion. The substantial care burden of caregivers who live with a relative with SMI has been shown to have a negative effect on their psychological health and social life and affects their sleep and work (Cheng et al., 2022).

The participants faced emotionally exhausting dilemmas, such as risking suicide or admitting him or her to specialized care. Such widespread dilemmas are known to be primary stressors in the caregivers of individuals with SMI (Boydell et al., 2014; Labrum & Solomon, 2018). The participants often had a justified fear of the safety of their family members, as well as of their own safety and that of others. Although the prevalence of violence is moderate in persons with SMI, rates are higher in first-episode psychosis, and those who are violent often target their families (Onwumere et al., 2018). Some of the crucial factors related to family violence found by Labrum and Solomon (2016) that also apply to our findings are co-residency, limit-setting practices, and ill relatives’ substance abuse. Substance use disorder in combination with SMI occurs frequently and often leads to violent behavior (Hunt et al., 2018; Patterson et al., 2021).

In a recent review of violence toward family caregivers, the authors concluded that better support services for persons with SMI could reduce their reliance on family members and thus decrease aggression (Labrum & Newhill, 2021). Furthermore, the authors suggested supporting family members to prevent and manage conflicts. In accordance with our results, researchers have reported that it is common for caregivers to emphasize their fear for the ill relative’s welfare while evading communication about their own safety issues (Klages et al., 2020; Onwumere et al., 2019). These researchers argued that professionals should routinely inquire about violence to ensure the safety of those at risk of harm.

Overall, the siblings’ daily lives seemed to be less dominated by emotional distress. Their ability to share their challenges as the main caregivers with the people they trust might have alleviated their distress and strengthened their resilience. Findings from the World Health Organization’s mental health surveys show that caring for children and spouses is more burdensome than caring for siblings, with female caregivers reporting a higher burden than men (Viana et al., 2013). The participating mothers described intense negative emotions using metaphors such as “bleeding inside” and “living in hell,” in contrast to the other relatives. These differences might be understood in light of differences in the caregivers’ perceived freedom of choice of engagement in the care of their family member (Zegwaard et al., 2013).

This could also be attributed to sex differences. Möller-Leimkühler and Wiesheu (2012) reported more negative emotions in female than in male caregivers, and Johansson et al. (2015) found that mothers’ health was more affected than fathers’ health. Our findings add to the findings of Landon et al. (2016), showing that mothers also expressed more concern about how their ill child influenced family functioning. Traditionally, women are more likely to be the main caregivers, to engage in more intense caregiving, and to report a higher caregiver burden (Pinquart & Sorensen, 2006). Nonetheless, Onwumere et al. (2021) showed that both female and male parents had similar levels of emotional distress and similar understanding of the illness, possibly reflecting increased gender equality.

Parents’ expressions of sadness and regret might reflect the complex process of adapting to the loss of loved ones as they had known them or had hoped they would become. The uncertain and shifting illness trajectories seem to leave these caregivers in a state of emotional limbo (Karp & Tanarugsachock, 2000), preventing some and delaying others from coming to terms with the loss and modifying their expectations of their struggling family members. These findings may be related to complicated grief as reported by Rachamim et al. (2022). Participants’ accounts of self-conscious emotions such as shame, guilt, and self-blame might also be understood as complicated grief reactions closely related to posttraumatic stress and depression (Duncan & Cacciatore, 2015; Kingston et al., 2016). Boss (2007) claimed that if a loss is ambiguous, it freezes the grief process and blocks coping and decision-making processes. Feelings of hopelessness, helplessness, and guilt may also occur (Boss, 2016).

Caregivers’ grief can also be seen as disenfranchising, as their loss is not socially acknowledged, making them hide their grief reactions (Doka, 2008). The losses of caregivers in our study can be perceived as both ambiguous and disenfranchised, as the ill person is still physically there but is perceived as not present psychologically due to major changes in personality (Boss, 2007; Doka, 2008). Caregivers’ negative emotions and hidden grief tend to lead to withdrawal from others, thus depriving them of opportunities for potential support and enhanced coping (Doka, 2008; Shiraishi & Reilly, 2022). Caregivers’ sense of guilt and shame might also be related to an overrated sense of responsibility in relation to self-harm and suicide (Hughes et al., 2017; McLaughlin et al., 2016). A better understanding of the psychological factors accounting for caregiver distress early in the illness trajectory might prevent long-term distress in caregivers and support loved ones’ recovery (Jansen et al., 2015b).

The narratives of our participants, especially those of the parents, revealed few positive experiences and hopes. Informal caregiving in families with SMI may encompass positive and life-changing experiences (Kitzmüller et al., 2023; Shiraishi & Reilly, 2019) in addition to promoting well-being and resilience among family caregivers (Chen & Lukens, 2011; Wiens & Daniluk, 2009). The emotional distress expressed by family caregivers in our study and the paucity of positive aspects in their narratives might reflect the fact that several of the ill family members had co-occurring substance use disorder and SMI, a combination known to be prevalent and challenging (Kingston et al., 2017). Onwumere et al. (2008) reported that family caregivers assessed caregiving more positively when they found that both the caregiver and the person with SMI had some control over their illness. Deficient emotional support might also account for the lack of positive accounts (Shiraishi & Reilly, 2019) and differences between parents and other caregivers.

## Study Limitations

Our study has some limitations. First, we used data from 2009 to 2010. These experiences might thus be less relevant today because of the implementation of new guidelines in many countries and the fact that healthcare policymakers and providers have increasingly recognized family support as essential (Hestmark et al., 2021; Mosse et al., 2022). Nonetheless, family support and involvement have not been systematically implemented (Hestmark et al., 2021) and hospital stays have become increasingly shorter. The effectiveness of increasingly available internet-based information and support for carers is yet to be established, although some researchers have found promising results (Kaewwanna et al., 2023). The first author’s clinical experience and feedback from colleagues and family caregivers suggest that the findings are highly relevant.

Second, a single interview was conducted with a different aim. Conducting several or even fresh interviews would most likely have provided new information due to individuals’ constantly transforming preunderstandings (cf. Fleming & Robb, 2019). Nonetheless, we argue that since the first author conducted the interviews in a dialogical manner and ensured a thorough understanding at that point in time (cf. Fleming et al., 2003), they provided a firm and reliable base for the secondary analysis. The aim of the present study resonated well with that of the primary study and the participants’ consent. Third, the sample size was small and the study was heterogeneous. We contend that the rich descriptions of relevant experiences from seven different families and three different family roles support and add to the prior research.

## Concluding Reflections

This study revealed that family caregivers of persons with SMI face multiple threats to their well-being and sometimes even their health due to the unpredictability of their life situation and emotional turmoil involving infinite vigilance and fear. The lack of individualized and timely information, opportunities to confide, and inclusion in care planning left them struggling with numerous challenges. These results provide additional insights into clinical practice and reveal issues that researchers should more closely examine in families with SMI, including caregivers’ experiences of trauma, threats of violence, and rejection.

## Implications for Practice and Research

These findings underline the importance of providing appropriate information, guidance, and emotional support to meet the needs of family caregivers as early as possible and as long as necessary. Communication skills training for healthcare professionals can improve relationships with family caregivers who need knowledge and encouragement to persevere. The relevance of a diagnostic communication protocol has already been tested in patients with serious mental illnesses (Milton & Mullan, 2017) and should be further developed to fit family caregivers.

Empathic approaches that explicitly address everyday functioning as caregivers, coping with long-term and recurring distress, safety issues, self-blame, and grief might enhance family caregivers’ positive emotions, mastery, and health. The specific needs of family members according to their different relationships and roles should be explored further. Policymakers should focus on implementing support programs that encompass the emotional needs of family caregivers with long and complicated trajectories, noting the need for caregiver-only interventions. Further research should address the lack of knowledge about family caregivers’ long-term trajectories in caring for individuals with SMI and their experiences with family interventions.
